# Cryoneurolysis: A Novel Treatment for Management of Spasticity. Presentation of a Case Series

**DOI:** 10.1177/27536351251340216

**Published:** 2025-07-15

**Authors:** Anton Pick, Rachel Dye, Melanie K. Fleming

**Affiliations:** 1 Oxford Centre for Enablement, Oxford University Hospitals NHS Foundation Trust, UK; 2 Wellcome Centre for Integrative Neuroimaging, FMRIB, Nuffield Department of Clinical Neurosciences, University of Oxford, UK

**Keywords:** cryoneurolysis, spasticity, pain, case reports, neurological conditions, goal attainment

## Abstract

**Background:**

Spasticity is a motor phenomenon occurring in disorders of the central nervous system that impacts on active and passive function, and quality of life. Pharmacological, physical and surgical management options are available, each of which have limitations. Cryoneurolysis is a technique developed for the treatment of pain which involves the controlled freezing and thawing of peripheral nerves. Recent case reports and series have suggested it may offer a novel treatment approach for pain associated with spasticity.

**Objectives:**

To report on the evaluation of cryoneurolysis in the first cohort of patients treated in a UK spasticity clinic.

**Methods:**

Eight patients with a variety of neurological conditions (aged 25-75 years) underwent cryoneurolysis. Each had been receiving regular botulinum toxin injections and had ongoing treatment goals. All patients first underwent diagnostic nerve blocks with local anaesthetic to determine their appropriateness for the treatment. Cryoneurolysis was then performed with ultrasound and nerve stimulator guidance. Assessments included goal attainment, Modified Ashworth Scale (MAS), ArmA, LegA and the patient reported impact of spasticity scale (PRISM), alongside patient satisfaction and side effect questionnaires. Assessments were at baseline and at regular intervals over 9 to 12 months.

**Results:**

All patients attained at least one of their goals, with sustained effect for more than 6 months. MAS demonstrated mixed or modest improvements. Functional outcome measures (ArmA/LegA) showed several meaningful improvements, particularly in passive function. There was an indication of an improvement in PRISM across domains, which plateaued at 6 months. Post-procedure pain was the most common side effect but subsided in all affected patients by 3 months. Patient satisfaction was positive.

**Conclusions:**

Our findings contribute to a growing base of case reports and series suggesting that cryoneurolysis could be a potentially useful treatment modality for spasticity. Future controlled studies should aim to evaluate cost-effectiveness and compare with existing treatments.

## Introduction

Spasticity is an umbrella term used to describe impairments caused by the dysregulation of muscle tone and control occurring in the context of damage or dysfunction in the central nervous system. It is estimated to occur in up to 80% of spinal cord injury patients,^1^ up to 39.5% of stroke patients with paresis^2^ and 84% of patients with multiple sclerosis.^3^ Spasticity can manifest in impairments such as pain and stiffness. It can substantially restrict activities and participation, such as by causing difficulties with personal care tasks and mobility, impacting on quality of life.^4^

Available treatments such as oral anti-spasmodics, botulinum toxin injections and physical therapies can provide relief for patients living with spasticity, but each has limitations.^5-7^ Botulinum toxin injections are now often considered a first line treatment for focal spasticity. However, they typically provide only temporary relief necessitating frequent repeat treatments (e.g. every 3-4 months). This places a significant burden on both patients and healthcare providers, involving substantial time commitment and treatment associated costs.

Cryoneurolysis involves the controlled freezing and thawing of nerve tissue aimed at disrupting its function.^8^ Since the 1970s, cryoneurolysis has primarily been used to target sensory nerves for the management of pain, but there is a growing interest in its potential application in the management of spasticity.^9^ Various cryoneurolysis devices have been developed, from desktop systems connected to external gas cannisters, to handheld wireless systems containing small replaceable gas cannisters. Several of these devices have been licensed for clinical use in the United Kingdom for the management of pain. All of the systems work by moving highly pressurised, liquified gas around a closed loop system, through a needle’s hollow lumen, to the needle tip. At the needle tip it undergoes rapid expansion into a lower pressure space, thereby, through action described by the Joule Thompson Effect, dropping the needle tip temperature towards the boiling point of the gas.^8^ The aim is to produce a sustained but reversible disruption to nerve function by causing axonotmesis at a temperature between −20°C and −100°C. Any warmer will either have no effect or produce a neuropraxia, resulting in only little or short-lived effects, and any colder, an irreversible neurotmesis may occur.^8^ Gases with boiling points within this target temperature range are carbon dioxide and nitrous oxide, both of which are used in devices currently marketed for use in cryoneurolysis. Animal studies have demonstrated that exposure of nerves to these temperatures does indeed result in axonotmesis.^10,11^ After this degree of nerve injury, gradual regeneration and remyelination with normalisation of structure and function occurs within weeks to months. Case reports suggest that cryoneurolysis can lead to immediate relaxation of the affected muscles, resulting in reduced pain as well as improved joint range of motion and enhanced functional mobility.^12,13^

Here we report an evaluation of the first cohort of patients treated as part of the introduction of cryoneurolysis for the management of spasticity related pain in an outpatient spasticity service in the United Kingdom.

## Materials and Methods

### Cohort

Eight patients underwent cryoneurolysis in a spasticity outpatient service. Patients ranged from 25 to 75 years of age and 3 were male. Diagnoses included stroke (n = 5), Cerebral Palsy (n = 1), Multiple Sclerosis with Progressive Multifocal Leukoencephalopathy (n = 1) and Hereditary Spastic Paraparesis (n = 1). All patients were well known to the service and had been previously treated with botulinum toxin but with only limited success and therefore had ongoing treatment goals. Patients deemed clinically suitable were only offered the treatment if they demonstrated capacity to consent for treatment. The consent process was informed by published literature exploring potential risks and benefits of the procedure from clinicians with experience using cryoneurolysis to manage pain and spasticity.^8,14^ Contraindications included Raynaud Syndrome, cryoglobulinaemia and cold urticaria. Risks explained during the treatment consent process included possible bleeding, bruising, infection, damage to the nerve or surrounding structures, persistent nerve pain, the potential for no or limited clinical effect, ‘frost bite’ and discolouration of the skin at the site of cryoneurolysis. All patients provided written consent for the treatment. There were no patients who were offered the treatment and declined it. Data were collected routinely as part of the established protocol of the clinical service. The project was approved by standard hospital governance processes as a service evaluation project and was therefore deemed not to require ethical approval. Data were handled in accordance with data protection protocols of the hospital and pseudonymised prior to analyses.

### Treatment Procedures

All patients initially underwent diagnostic nerve blocks using 1% Lidocaine, to target nerves or nerve branches. Nerve localisation was carried out using an ultrasound and nerve stimulator. This approach mirrors that of published cases to date. Nerve blocks with local anaesthetic provide the patient and clinician with a temporary demonstration of the potential effects of cryoneurolysis. This informs the patient’s appropriateness for cryoneurolysis and supports the consenting procedure.

Patients deemed suitable went on to have cryoneurolysis on the same day. Additional 1% lidocaine was used to anaesthetise the skin for insertion of a needle introducer (a 16G cannula) for the cryoprobe. The cryoprobe was then introduced to the target nerve or nerve branch. One or more 110 second cycles of freezing and thawing were used for each nerve or nerve branch.

### Assessments

Patients were assessed at baseline (pre-treatment) and at 3 and 6 months post treatment. If treatment effects were found to be waning at 6 months, patients were reviewed again at 9 months (n = 3). If treatment effects were sustained at 6 months, patients were reviewed at 12 months only (n = 5). The outcome measures/scores at the ‘final timepoint’ were therefore either at 9 or 12 months. Additionally, patients were contacted at 1 to 3 days post procedure and again at 4 weeks to check for adverse effects.

Patient goals were set at baseline and reviewed at each follow-up appointment using the global assessment of benefit (GAB) rating scale.^15,16^ This is a simple numerical scale, which can be used to rate the effects of a treatment and to quantify goal attainment. It is scored as −2 Much worse; −1 Worse; 0 The same; +1 A bit better or +2 Much better.

Spasticity was assessed using the Modified Ashworth scale (MAS). The MAS is a commonly used clinical and research assessment tool for the measurement of spasticity.^17^

Patients also completed the Patient Reported Impact of Spasticity Scale (PRISM) questionnaire^18,19^ at each assessment timepoint. There are 7 domains in the PRISM including social avoidance/anxiety, psychological agitation, daily activities, need for assistance/positioning, social embarrassment, need for intervention and positive impact. In all domains apart from positive impact, higher scores represent greater severity.

For patients who received upper limb treatment, the ArmA questionnaire was completed^20^ and for those who received lower limb treatment, the LegA questionnaire was completed.^21^ The ArmA and LegA incorporate several domains including active function, passive care and quality of life (LegA only). The higher the score, the greater the impairment. A clinically important change in both ArmA and LegA is represented by a 3 point change in scores.

Finally, a patient experience questionnaire was administered which included a 0 to 10 numerical rating scale of satisfaction.

## Results

### Goals

There were 24 goals across the patients (range 2 to 4 goals per patient), which broadly related to upper limb passive care (5 goals), upper and/or lower limb pain (5 goals) and function/participation/movement (14 goals). Goal attainment using the global assessment of benefit (GAB) scale, at 3, 6 months and final time points post-treatment is depicted in Table 1. All patients achieved at least one of their goals. All upper limb goals relating to passive care, pain on movement and associated reactions were achieved. Only 2 out of 5 goals relating to upper limb function/participation were achieved. The 3 goals not achieved were all for the same patient, including ‘improve ability to open hand without using the other hand’, ‘to be able to hold a knife’ and ‘to be able to hold a kettlebell’. All goals related to lower limb movement and walking were achieved. Limited goal data were available at the final time point, but of the 14 goals that were rated, all were identified to have been achieved.

**Table 1.** Goals scored as −2, −1, 0, +1 or +2 using the global assessment of benefit (GAB) at each assessment timepoint.

Categories	Number of goals	Score at 3 months post-treatment					Score at 6 months post-treatment					Score at final timepoint post-treatment (9 or 12 months)				
		−2	−1	0	+1	+2	−2	−1	0	+1	+2	−2	−1	0	+1	+2
UL – passive care tasks	5	0	0	0	1	4	0	0	0	1	4	0	0	0	0	5
UL – pain on movement	4	0	0	0	1	3	0	0	0	1	3	0	0	0	0	4
UL – Associated reaction of EF	2	0	0	0	1	1	0	0	0	0	2	0	0	0	0	2
UL – function/participation	5	0	0	3	0	2	0	1	2	0	2	0	0	0	0	3
LL – Movement and walking	7	0	0	0	0	7	0	0	0	0	7	0	0	0	2	4
LL – pain	1	0	0	0	0	1	0	0	0	0	1	0	0	0	0	1

Not all goals were scored at final timepoint.

### Spasticity Measures

Six patients received cryoneurolysis treatment to their lower limbs. The nerves treated and the MAS scores for the corresponding muscles at each time point are shown in Table 2. Results were variable but show modest improvements in a majority of the cases. The MAS was reduced for at least 6 months in 13 of 16 spastic muscles targeted with cryoneurolysis treatment. Three of 6 patients had complete resolution of spasticity on MAS in triceps surae muscles, sustained at final follow-up at 9 to 12 months. Several muscles showed no clear change in MAS after treatment.

**Table 2.** Lower limb modified Ashworth scores for each patient treated.

Nerves treated	Muscle	Baseline	3 months	6 months	Final time point (9-12 months)
Tibial trunk	Soleus	1+	0	1	0
	Gastrocnemius	1+	0	1	0
	Tibialis posterior	1+	1	1	1
Tibial trunk	Soleus	2	no data	0	0
	Gastrocnemius	2	no data	0	0
	Tibialis posterior	1	no data	1	1
Branches of tibial nerve to soleus, gastrocnemius and tibialis posterior	Soleus	1	0	0	0
	Gastrocnemius	1	0	0	0
	Tibialis posterior	1+	1	1	1+
Tibial trunk	Soleus	1+	1+	1	1+
	Gastrocnemius	1+	1+	1	1
Femoral nerve	Quadriceps	no data	1	1	1
Tibial trunk	Soleus	1+	1	0	no data
	Gastrocnemius	1	1+	1	no data
	Tibialis posterior	1	1+	1+	no data
Branches of tibial nerve to gastrocnemius and soleus	Soleus	1+	no data	1	1
	Gastrocnemius	1+	1	1	1
Bilateral obturator nerves	Hip adductors	1+	0	1+	1+

Six patients received cryoneurolysis treatment to their upper limbs (note: some patients had both upper and lower limbs treated). MAS scores at each time point are shown in Table 3. The findings across the cases are less consistent than those in lower limbs. A sustained reduction on MAS for up to 6 months was shown in 9 of 21 spastic muscles targeted. Several muscles showed no or minimal changes in MAS.

**Table 3.** Upper limb modified Ashworth scores for each patient treated.

Nerves treated	Muscle	Baseline	3 months	6 months	Final time point (9-12 months)
Medial and lateral pectoral nerves	Pectorals	No data	1	1	1+
Musculocutaneous branch to brachialis	Elbow flexors	1	0	0	1
Medial and lateral pectoral nerves	Pectorals	1+	1	1	1
Musculocutaneous nerve branch to biceps and brachialis	Elbow flexors	1+	1+	1+	1+
Ulnar nerve branches to FCU	Wrist flexors	1	1+	1+	1+
Medial and lateral pectoral nerve	Pectorals	No data	No data	No data	No data
Ulnar nerve and median nerve	Lumbricals	4	1	1+	1
	Finger flexors (FDS)	1	0	0	1
Lateral and medial pectoral nerve	Pectorals	No data	0	1	No data
Latissimus dorsi	IR of shoulder	No data	1	No data	No data
Musculocutaneous to biceps	Elbow flexors	2	1	1	1+
Ulnar nerve	Wrist flexors	1+	1	1	1+
	Lumbricals	3	0	1+	1+
	FDS/FDP	0	0	0	0
Lateral and medial pectoral nerve	Pectorals	No data	1	1	1+
Single branch to biceps and brachialis	Elbow flexors	1+	1+	1+	1+
Median nerve	Finger flexors (FDS)	2	1	1+	1+
	Finger flexors (FDP)	1	1	1	0
Lateral and medial pectoral nerve branch	Pectorals	No data	No data	1	1
Musculo-cutaneous nerve – biceps branch	Elbow flexors	1+	0	1	1+
Median nerve	Finger flexors (FDS)	1+	1	1+	2
	Finger flexors (FDP)	1	1	1	1+

### Function and Passive Care Questionnaires

**Figure 1.**
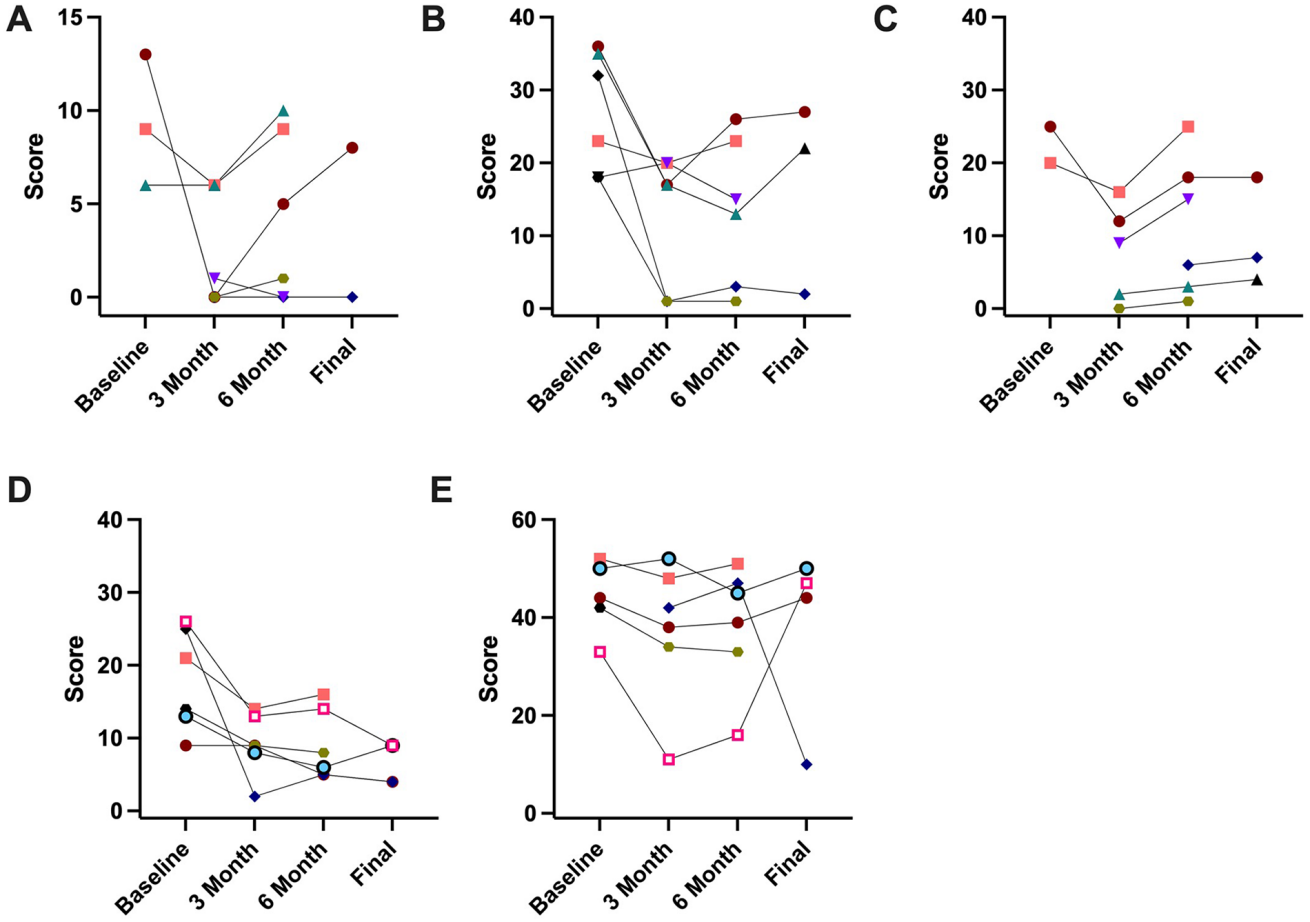
Top Panel: LegA score over time for individual patients, A = passive care, B = active function, C = quality of life. Bottom Panel: ArmA score over time for individual patients. D = passive care, E = active function. Lower numbers indicate better function or quality of life.

The LegAA and ArmA questionnaires were used with patients with relevant goals. All Individual scores are demonstrated in Figure 1 and supplementary tables S1 and S2.

### LegA

Only 3 patients completed section A (passive care) at baseline. Two of the 3 experienced a clinically important improvement (3 point reduction) at 3 months post-treatment, which was maintained for 1 patient at 6 and 12 months.

Six patients completed section B (active movements) at baseline, and 5 patients experienced a clinically meaningful improvement at 3 months post-treatment. This improvement was maintained for 3 patients. One patient’s score worsened at 6 and 12 months compared to 3 months, but it remained better than at baseline. One patient had an initial worsening, but an improvement at the 6-month assessment.

Only 2 patients completed section C (impact/participation) at baseline, both of whom experience a clinically meaningful improvement at 3 months which then worsened at 6 months (remaining better than baseline for one).

### ArmA

Six patients completed section A (passive care) at baseline, 5 of whom experienced a clinically important improvement (3 point reduction) at 3 months post-treatment, which persisted at 6 months. One patient had no improvement at 3 months but had a meaningful improvement at 6 months which persisted till 12 months.

Five patients completed section B (active movements) at baseline. Four patients experienced a clinically important improvement at 3 months, and this persisted at 6 months for 3 of them. One patient experienced an initial improvement, but at 12 months their score was worse than at baseline.

### Impact of Spasticity on Quality of Life (PRISM) Questionnaire

**Figure 2.**
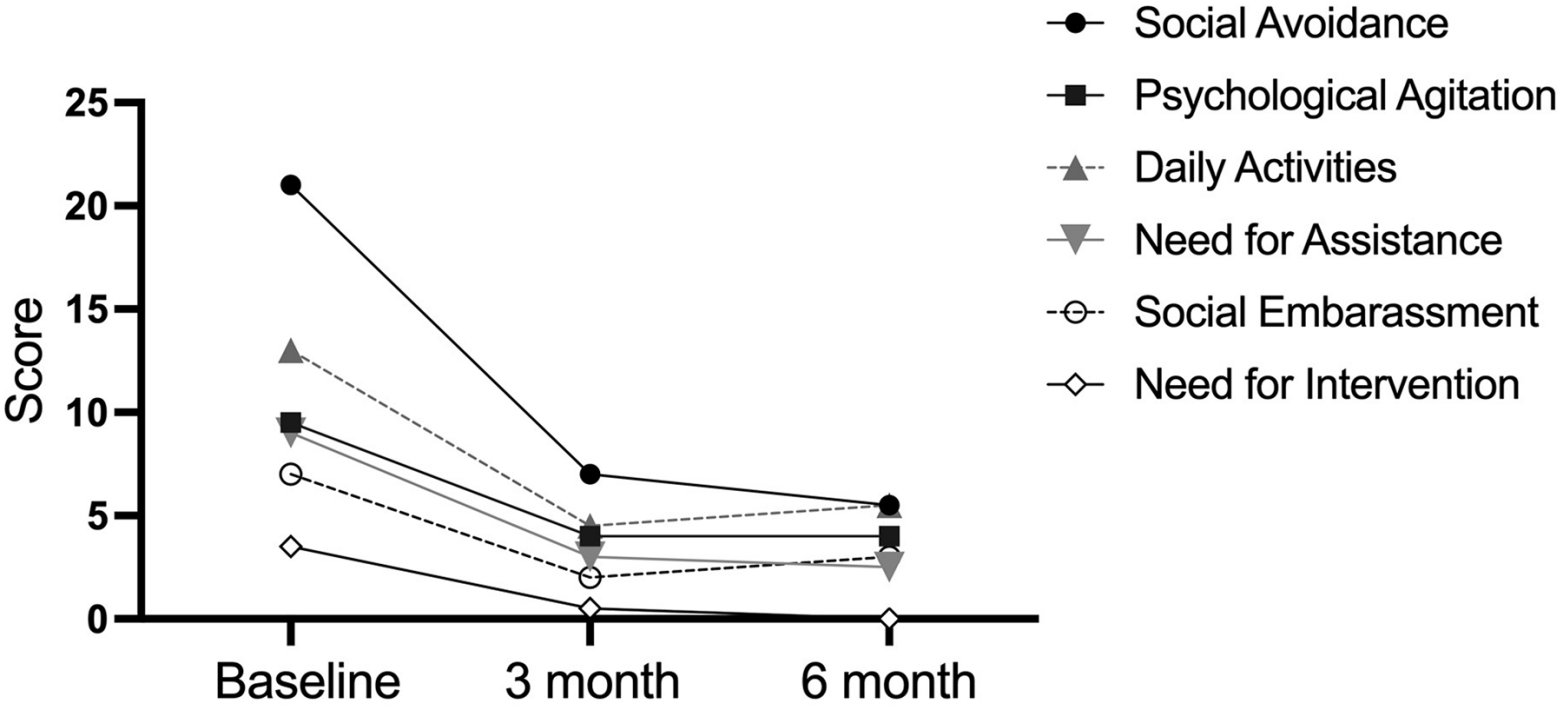
Median score across patients for each subcomponent of the PRISM. Lower values indicate less impact of spasticity.

The completion rate for the PRISM questions varied across domains and timepoints. We excluded data from the final assessment, as only 5 patients filled in the questionnaire. For the remaining timepoints, we calculated the median across the 8 participants (Figure 2 and supplementary table S3). Domain 5 (positive) was excluded as the questions contributing were inconsistently completed. Overall, there is an indication towards an improvement at a group level from baseline to 3 months across domains, and the appearance of plateau in score at 6 months.

### Adverse Reactions

Potential adverse reactions including pain, bleeding and bruising were discussed with the patients as part of the consent process (Table 4). As per routine care in the service, patients were given contact details for the department should they want to discuss any complications. Management of complications was informed by published literature on the use of cryoneurolysis in the management of pain and spasticity.^8^ Six out of 8 patients reported some general pain related to the procedure, lasting from a few days to 2 weeks. Three out those 6 patients then developed neuropathic pain 1 to 2 weeks post procedure, which required additional treatment with pregabalin. One patient experienced significant foot and leg pain, after treatment to the tibial nerve trunk; 1 patient experienced neuropathic pain in her medial forearm and palm, after treatment to branches of her musculocutaneous nerve and median nerve; and 1 patient experienced neuropathic pain in the lateral forearm, palm and leg, following treatment to median, ulnar and tibial nerve trunks. At the 3 month follow up, all pain had resolved and pregabalin was either stopped or decreased to original dosage.

**Table 4.** Reports of adverse reactions at each assessment timepoint.

Adverse reaction	<1 month	3 months	6 months	Final timepoint
Pain post procedure	6	None	None	None
Persistent pain requiring additional treatment	3	Resolved	None	None
Bruising at site	2	None	None	None
Numbness	3	3	1	1

Two patients reported some bruising at the insertion sites after the treatment, which resolved quickly.

Three patients reported numbness. Forearm numbness was reported by 1 patient after treatment to branches of the musculocutaneous nerve, which resolved by 6 months; right leg knee to shin numbness was reported by 1 patient, after treatment to the obturator nerve, which remained at 6 months but resolved by 12 months; and 1 patient reported numbness in her little finger, following treatment to the ulnar nerve, which persisted at 12 months.

### Patient Satisfaction

At 6 months and final assessment timepoint (9 or 12 months), patients were asked to complete a patient satisfaction questionnaire that included a visual analogue scale to grade their overall satisfaction with the treatment and a question as to whether they would recommend the treatment to others. All patients completed the questionnaire at 6 months post treatment and 5 out of the 8 patients completed it again at 12 months. At 6 months, 7 patients rated their satisfaction as 10/10 and 1 patient rated it as 9/10. Seven out of the 8 patients stated they would recommend the treatment to others (one questionnaire was incomplete and some questions remained unanswered). At 12 months, 4 out of the 5 patients rated their satisfaction as 10/10, one rated it as 8/10, and all stated they would recommend the treatment to others.

## Patient Narratives

### Ms X

Ms X is a 75 year old female who had a right basal ganglia stroke in 2020. She presented with significant left ankle inversion and an associated reaction in her left elbow flexors when walking and standing. The ankle inversion limited her mobility such that she was unable to take a single step when not wearing her rigid ankle-foot orthosis. The associated reaction of her left elbow flexors impacted on her balance. Ms X had received regular Botulinum toxin injections in the outpatient spasticity service for over 2 years, with limited effect.

Ms X’s treatment goals were to

- Reduce elbow flexion when walking
- To improve foot contact with the floor to help stability in walking
- To reduce pain and discomfort on shoulder movement

Following positive results of diagnostic nerve blocks, Ms X underwent cryoneurolysis to her lateral and medial pectoral nerves, musculocutaneous nerve branch to brachialis and her tibial nerve trunk. The procedure was well tolerated and there was an immediate reduction in ankle inversion and the associated reaction of her elbow flexors. Immediately post treatment, Ms X was able to walk for the first time since her stroke without needing her ankle-foot orthosis.

Two weeks post treatment, Ms X developed significant neuropathic pain around her foot and calf that disturbed her sleep and limited her ability to weight bear on the affected leg. She temporarily moved in with her daughter to get additional care support. At its worst she rated the pain at 10/10 severity. She was started on twice daily Pregabalin which helped to ease the pain down to 5/10 severity after 1 week. After 2 weeks, Ms X omitted the day time pregabalin dose and only required 50 mg at night to relieve the pain. By 3 months, Ms X had weaned off the pregabalin entirely.

Despite the post treatment neuropathic pain, Ms X rated her satisfaction with the procedure as 10/10 in the patient satisfaction questionnaire. All goals were rated at +2 Much Better on the GAB, at 12 months. At 6 months she wrote ‘foot flat on floor, ankle turns over less, ankle more flexible, improved walking’ on her patient satisfaction questionnaire and again at 12 months her comments were ‘foot flat on floor, improved mobility’.

Given ongoing and stable benefits of the cryoneurolysis at 12 months, Ms X was discharged from routine follow up in the spasticity service.

### Mr Y

Mr Y is a 60 year old man who had a right middle cerebral artery stroke in 2015. He presented with painful toe clawing, which impacted on his balance and mobility; shoulder pain with movement, affecting daily care tasks; and severe pain and tightness in the hand and wrist making hand hygiene and care difficult. The pain and tightness in his hand was so significant, he had to have his wedding ring cut off after his stroke and could no longer hold hands with his wife.

He had been seen regularly in our outpatient spasticity service and received botulinum toxin injections for his left arm and leg for over 5 years. Mr Y was then listed for tendon lengthening surgery and hyperselective neurectomy to median nerve branch to finger flexor muscles.

Mr Y’s treatment goals were;

- To feel more stable when walking
- To be able to passively open the fingers of the left hand with less pain
- To be able to raise left hand to mouth
- To improve pain-free movement of the shoulder

After positive results from diagnostic nerve blocks, cryoneurolysis was performed on his medial and lateral pectoral nerves, ulnar nerve, median nerve and tibial nerve trunk. Immediately post treatment, the pain and tightness of his hand reduced so much that he was able to put his wedding ring back on and managed to passively open his hand sufficiently to hold hands with his wife for the first time in 9 years. For his leg, the painful toe clawing reduced and he gained active ankle dorsiflexion, which improved his walking and stability.

Within 2 weeks, Mr Y developed neuropathic pain on the lateral cutaneous skin of his forearm, over his palm and on his heel. To manage the pain, his dose of pregabalin was increased and he was also prescribed a lidocaine patch. By 3 months, all pain had resolved and he was able to wean the pregabalin dose to the original level.

Mr Y rated his satisfaction with the procedure as 9/10 on the patient satisfaction questionnaire. All goals were self-rated as +2, Much better on the GAB at 3-, 6- and 9-month follow-up appointments. At 6 months, there was some waning of effects for the upper limb and so Mr Y was reviewed again at 9 months. Mr Y chose to cancel his planned upper limb surgery and repeat cryoneurolysis was arranged instead.

### Mr Z

Mr Z is a 50 year old male who suffered a right middle cerebral artery stroke in 2019. He had received regular botulinum toxin injections in the outpatient spasticity service for over 3 years, with limited benefit. He presented with tightness and pain of his hand and fingers, making hand hygiene and splint wear difficult, an associated reaction of his elbow flexors and impaired mobility. His treatment goals were:

- To be able to walk without the use of the ankle brace
- To be able to apply the hand splint more easily
- Improve comfort and ease of opening hand
- To be able to grasp pulley with left hand to complete upper limb exercises

After positive results from diagnostic nerve blocks, Mr Z underwent cryoneurolysis to his lateral and medial pectoral nerve, musculocutaneous nerve branches to biceps and brachialis, median nerve, tibial nerve and femoral nerve branch to rectus femoris. Mr Z experienced a few weeks of intermittent neuropathic pain, but did not require any additional prescribed analgesics. The treatment was immediately successful, and had a significant impact on his quality of life. His goals were all rated as +2 ‘much better’ on the GAB at each follow-up time point and he rated his satisfaction with the procedure as 10/10. On the patient satisfaction questionnaires, he wrote ‘I have never been more confident as I am since stroke four years ago’ and ‘having my best life at the moment at home and at work.’ The benefits started waning at around 10 and a half months and at 12 months repeat cryoneurolysis was arranged.

## Discussion

The current paper presents clinical outcome data for the first 8 patients in the UK treated with cryoneurolysis for the management of spasticity and followed up over 9 to 12 months. In this cohort, patients showed sustained goal attainment, particularly for passive care goals. Although there is no control group, and no statistical comparisons performed, there appeared to be an improvement at the group level in several outcomes. Improvements in passive function are suggested by both ArmA and LegA results. The patient reported impact of spasticity (PRISM) questionnaire showed notable reduction in the negative impacts of spasticity after cryoneurolysis in all domains, sustained up to at least 6 months. At an impairment level, Modified Ashworth Scale changes were modest. Given the other more positive findings in the cohort, this limited change in MAS may reflect limitations in the sensitivity of the scale, rather than lack of treatment effect.^22^ Future research should include additional measures of spasticity, such as the Modified Tardieu Scale as well as electromyography assessments.

The most common adverse effects were post-procedure pain and subsequent neuropathic pain lasting several weeks. This was typically treated with pregabalin. By 3 months post-treatment, all pain had resolved and pregabalin had either been stopped or decreased to its original dosage. This is consistent with reports from Winston et al^14^ that pain typically resolve within 3 months of treatment. Nevertheless, future studies will need to continue to systematically monitor adverse effects and explore ways to mitigate them.

Some data were missing in the case series. Whilst goal attainment was recorded for all patients at baseline and at every follow-up point, Modified Ashworth Scale scores and patient reported outcome measures including LegA, ArmA and PRISM were less reliably collected. Unlike prospective research studies, this was a real-world, descriptive case series relying on data recorded within existing records and this varied in completeness. The goal of the series was to illustrate patterns and highlight observations, rather than to draw conclusions based on statistical analyses. Reasons for incomplete data are likely several-fold, but the time burden of completing lengthy patient reported measures in the context of a busy clinic setting probably contributed. Modified Ashworth Scale scoring at follow-up was also likely limited due to the short duration of follow-up appointments. Despite the missing data, the core clinical findings remain interpretable and consistent across cases.

These findings contribute to a growing literature base of uncontrolled studies that suggest a potential role of cryoneurolysis in the management of spasticity. However, it must be noted that, to our knowledge, no randomised controlled studies have been completed to date. Despite some variability in outcomes and the pain reported by several patients in this cohort after the procedure, all patients were positive about their experience of the treatment.

### Future Research

Assurance about the safety of cryoneurolysis can be drawn from over 40 years of its use in the management of pain.^8^ In addition, several animal studies have demonstrated that cryoneurolysis, at the temperatures thought to be reached by the devices currently available, causes reversible nerve injury or axonotmesis, with normalisation of nerve structure and function over time.^11^. However, in order to determine the role that cryoneurolysis should play in the multi-modal management of spasticity, several important questions must still be empirically answered in future studies.

#### How Should the Pain Occurring During the Procedure be Managed?

In the cohort described herein, patients received a diagnostic nerve block with local anaesthetic on the same day as the cryoneurolysis procedure. As such, pain during the procedure was limited. However, in routine practice, some clinicians do not anesthetise the nerve prior to cryoneurolysis. Anaesthetising the nerve removes the potential to stimulate it with the cryoprobe to confirm optimal probe position next to the nerve. However, without local anaesthetic, cryoneurolysis can be painful for patients.

#### What is the Rate, Duration and Impact of Post-Procedure Pain, and to What Degree is its Occurrence Modifiable Using Different Treatment Approaches?

Evidence from animal models suggest a correlation between partial neurolysis and subsequent hyperalgesia.^23^ Therefore, to avoid partial neurolysis, some clinicians suggest increasing the number and/or duration of freeze-thaw cycles, particularly for larger nerves. However, the ‘right’ number and duration of cycles for a given nerve is not yet known. In addition to the parameters of the treatment cycles themselves, nerve selection may also play a role in determining risk of post procedure pain. Although evidence is inconsistent, some studies of chemoneurolysis with phenol or alcohol suggest an increased risk of dysaesthesia with treatment of mixed motor sensory nerves. Chemoneurolysis and cryoneurolysis appear to cause different types of nerve injury. However, it is reasonable to hypothesise that targeting motor nerve branches and motor points may reduce the chance of causing post-procedure pain. However, it is the experience of the authors that at least transient neuropathic pain can occur after the treatment of both motor branches and mixed motor-sensory nerves, perhaps related to the presence of sensory afferents within what are generally thought to be ‘pure motor’ nerves.^24^ Optimal nerve targets for cryoneurolysis that provide a clinically significant duration of effect with minimal side effects need to be formally studied.

#### When Post-Cryoneurolysis Pain Does Occur, How Is It Optimally Managed?

The nature of any pain occurring post procedure must be determined prior to considering appropriate management. Soft tissue pain, such as due to over-stretch in the presence of diminished tone, and neuropathic pain have both been anecdotally reported. Validated assessment tools such as DN4 can be used to determine whether post-procedure pain is neuropathic in nature.^25^ The authors have encountered a mixture of soft tissue and neuropathic pain in patients treated with cryoneurolysis. If neuropathic pain does occur, published and anecdotal reports suggest the use of a neuropathic pain agent such as a gabapentinoid.^8,14^ The authors have found this to be an effective approach for most post-procedure pain. If the pain is severe and persistent, clinicians using cryoneurolysis for the management of pain have suggested repeating the cryoneurolysis, on the assumption that the pain is a result of incomplete neurolysis. Others suggest using long-acting local anaesthetic blocks (e.g. bupivacaine or ropivacaine) with or without a steroid. There are no studies on which to base clinical practice recommendations.

#### How Does Cryoneurolysis Compare to Current Standards of Care, in Terms of its Efficacy, Cost Effectiveness and Resource Burden, and How Does It Fit Within the Multimodal Set of Treatment Options?

As reported above, there are no published controlled studies of cryoneurolysis in the treatment of spasticity. Reasonable comparators for future controlled studies include botulinum toxin, given its widespread use as a first line treatment, surgical neurectomy and chemoneurolysis with Phenol or Alcohol. This case series, supported by published observational studies, suggest that cryoneurolysis may result in a longer duration of effect when compared with botulinum toxin injections but this needs formal evaluation in a controlled study. The cost effectiveness of cryoneurolysis must also be investigated.

### Summary

Here we demonstrate the potential utility of cryoneurolysis for the treatment of spasticity in a small cohort of patients with a variety of neurological conditions. Evaluation of routine clinical data suggests that responses were typically positive, with all patients achieving at least one of their goals. Despite several outstanding questions which require controlled trials in the future, cryoneurolysis appears to represent a potentially positive development in the often slow-changing field of neurological rehabilitation.

## Supplemental Material

sj-docx-1-rpo-10.1177_27536351251340216 – Supplemental material for Cryoneurolysis: A Novel Treatment for Management of Spasticity. Presentation of a Case Series Supplemental material, sj-docx-1-rpo-10.1177_27536351251340216 for Cryoneurolysis: A Novel Treatment for Management of Spasticity. Presentation of a Case Series by Anton Pick, Rachel Dye and Melanie K. Fleming in Advances in Rehabilitation Science and Practice

## References

[1] Sangari S Perez MA. Prevalence of spasticity in humans with spinal cord injury with different injury severity. J Neurophysiol. 2022;128:470-479. doi:10.1152/jn.00126.2022 35507475 PMC9423778

[2] Zeng H Chen J Guo Y Tan S. Prevalence and risk factors for spasticity after stroke: a systematic review and meta-analysis. Front Neurol. 2020;11:616097. doi:10.3389/fneur.2020.616097 33551975 PMC7855612

[3] Bethoux F Marrie RA. A cross-sectional study of the impact of spasticity on daily activities in multiple sclerosis. Patient. 2016;9:537-546. doi:10.1007/s40271-016-0173-0 27154536

[4] Milinis K Young CA. Systematic review of the influence of spasticity on quality of life in adults with chronic neurological conditions. Disabil Rehabil. 2016;38:1431-41. doi:10.3109/09638288.2015.1106592 26713898

[5] Jacinto J Varriale P Pain E Lysandropoulos A Esquenazi A. Patient perspectives on the therapeutic profile of botulinum neurotoxin type a in spasticity. Front Neurol. 2020;11:388. doi:10.3389/fneur.2020.00388 32477251 PMC7233119

[bibr6-27536351251340216] Lindsay C Kouzouna A Simcox C Pandyan AD. Pharmacological interventions other than botulinum toxin for spasticity after stroke. Cochrane Database Syst Rev. 2016;2016:10362. doi:10.1002/14651858.CD010362.pub2 PMC6457886 27711973

[7] Khan F Amatya B Bensmail D Yelnik A. Non-pharmacological interventions for spasticity in adults: an overview of systematic reviews. Ann Phys Rehabil Med. 2019;62:265-273. doi:10.1016/j.rehab.2017.10.001 29042299

[8] Gabriel RA Seng EC Curran BP Winston P Trescot AM Filipovski I. A narrative review of ultrasound-guided and landmark-based percutaneous cryoneurolysis for the management of acute and chronic pain. Curr Pain Headache Rep. 2024;28:1097-1104. doi:10.1007/s11916-024-01281-z 38963513 PMC11461560

[9] Winston P Vincent D. Cryoneurolysis as a novel treatment for spasticity, associated pain and presumed contracture. Adv Rehabil Sci Pract. 2024;13:13. doi:10.1177/27536351241285198 PMC11457193 39377080

[10] Shah SB Bremner S Esparza M , et al. Does cryoneurolysis result in persistent motor deficits? A controlled study using a rat peroneal nerve injury model. Reg Anesth Pain Med. 2020;45:287-292. doi:10.1136/rapm-2019-101141 32001625

[11] Hsu M Stevenson F-F. Reduction in muscular motility by selective focused cold therapy: a preclinical study. J Neural Transm. 2014;121:15-20. doi:10.1007/s00702-013-1077-y 23917804 PMC3889817

[12] Boissonnault È MacRae F Hashemi M Bursuc A Winston P . Cryoneurolysis of the femoral nerve for focal spasticity in an ambulatory patient. Arch Rehabil Res Clin Transl. 2024;6:100319. doi:10.1016/j.arrct.2024.100319 38482108 PMC10928293

[13] David R Hashemi M Schatz L Winston P. Multisite treatment with percutaneous cryoneurolysis for the upper and lower limb in long-standing post-stroke spasticity. Eur J Phys Rehabil Med. 2024;60:793-797. doi:10.23736/S1973-9087.24.08346-1 39007785 PMC11561588

[14] Winston P MacRae F Rajapakshe S Morrissey I Boissonnault È Vincent D , et al. Analysis of side effects of cryoneurolysis for the treatment of spasticity. Am J Phys Med Rehabil. 2023;102:1008-1013. doi:10.1097/PHM.0000000000002267 37104641

[15] Turner-Stokes L Fheodoroff K Jacinto J Maisonobe P. Results from the Upper Limb International Spasticity Study-II (ULIS-II): a large, international, prospective cohort study investigating practice and goal attainment following treatment with botulinum toxin A in real-life clinical management. BMJ Open. 2013;3:e002771. doi:10.1136/bmjopen-2013-002771 PMC3686177 23794582

[16] Royal College of Physicians. Spasticity in adults: management using botulinum toxin. National guidelines. RCP; 2018.

[17] Meseguer-Henarejos A-B Sánchez-Meca J López-Pina J-A Carles-Hernández R. Inter- and intra-rater reliability of the Modified Ashworth Scale: a systematic review and meta-analysis. Eur J Phys Rehabil Med. 2018;54:576-590. doi:10.23736/S1973-9087.17.04796-7 28901119

[18] Cook KF Teal CR Engebretson JC , et al. Development and validation of Patient Reported Impact of Spasticity Measure (PRISM). J Rehabil Res Dev. 2007;44:363. doi:10.1682/JRRD.2006.04.0036. 18247233 PMC2746440

[19] Ayoub S Smith JG Cary I , et al. The positive and the negative impacts of spasticity in patients with long-term neurological conditions: an observational study. Disabil Rehabil. 2021;43:3357-3364. doi:10.1080/09638288.2020.1742803 32223455

[20] Ashford S Turner-Stokes L Siegert R Slade M. Initial psychometric evaluation of the Arm Activity Measure (ArmA): a measure of activity in the hemiparetic arm. Clin Rehabil. 2013;27:728-740. doi:10.1177/0269215512474942. 23426566

[21] Ashford SA Siegert RJ Williams H Nair A Orridge S Turner-Stokes L. Psychometric evaluation of the leg activity measure (LegA) for outcome measurement in people with brain injury and spasticity. Disabil Rehabil. 2021;43:976-987. doi:10.1080/09638288.2019.1643933. 31328963

[22] Kumar RTS Pandyan AD Sharma AK . Biomechanical measurement of post-stroke spasticity. Age Ageing. 2006;35:371-375. doi:10.1093/ageing/afj084 16675479

[23] Myers RR Heckman HM Powell HC. Axonal viability and the persistence of thermal hyperalgesia after partial freeze lesions of nerve. J Neurol Sci. 1996;139:28-38. 8836969

[24] Adidharma W Khouri AN Lee JC , et al. Ensory nerve regeneration and reinnervation in muscle following peripheral nerve injury. Muscle Nerve. 2022;66:384-396. doi:10.1002/mus.27661. 35779064

[25] Aho T Mustonen L Kalso E Harno H. Douleur Neuropathique 4 (DN4) stratifies possible and definite neuropathic pain after surgical peripheral nerve lesion. Eur J Pain. 2020;24:413-422. doi:10.1002/ejp.1498 31660676

