# Supplementary material for: Cryoneurolysis: A Novel Treatment for Management of Spasticity. Presentation of a Case Series
Source: Adv Rehabil Sci Pract. 2025 Jul 15;14:27536351251340216. doi: 10.1177/27536351251340216 (PMC12264415; doi:10.1177/27536351251340216)
Supplement: sj-docx-1-rpo-10.1177_27536351251340216 – Supplemental material for Cryoneurolysis: A Novel Treatment for Management of Spasticity. Presentation of a Case Series [file sj-docx-1-rpo-10.1177_27536351251340216.docx]

**Supplementary Table S1.** Individual scores for LEG-A assessment at each timepoint (each row represents an individual patient)

| **Subsection A (passive care)** | | | | **Subsection B (active function)** | | | | **Subsection C (quality of life)** | | | |
| --- | --- | --- | --- | --- | --- | --- | --- | --- | --- | --- | --- |
| **Baseline** | **3 month** | **6 month** | **Final** | **Baseline** | **3 month** | **6 month** | **Final** | **Baseline** | **3 month** | **6 month** | **Final** |
| 13 | 0 | 5 | 8 | 36 | 17 | 26 | 27 | 25 | 12 | 18 | 18 |
| 9 | 6 | 9 |  | 23 | 20 | 23 |  | 20 | 16 | 25 |  |
| 6 | 6 | 10 |  | 35 | 17 | 13 | 22 |  | 2 | 3 | 4 |
|  | 0 | 0 | 0 | 32 | 1 | 3 | 2 |  |  | 6 | 7 |
|  | 0 | 1 |  | 18 | 1 | 1 |  |  | 0 | 1 |  |
|  | 1 | 0 |  | 18 | 20 | 15 |  |  | 9 | 15 |  |

**Supplementary Table S2.** Individual scores for ARM-A assessment at each timepoint (each row represents an individual patient)

| **Subsection A (passive care)** | | | | **Subsection B (active function)** | | | |
| --- | --- | --- | --- | --- | --- | --- | --- |
| **Baseline** | **3 month** | **6 month** | **Final** | **Baseline** | **3 month** | **6 month** | **Final** |
| 9 | 9 | 5 | 4 | 44 | 38 | 39 | 44 |
| 21 | 14 | 16 |  | 52 | 48 | 51 |  |
| 25 | 2 | 5 | 4 |  | 42 | 47 | 10 |
| 14 | 9 | 8 |  | 42 | 34 | 33 |  |
| 13 | 8 | 6 | 9 | 50 | 52 | 45 | 50 |
| 26 | 13 | 14 | 9 | 33 | 11 | 16 | 47 |

**Supplementary Table S3.** Individual scores for PRISM score at each timepoint (each row represents an individual patient)

| **PRISM subscale** | **Baseline** | **3 month** | **6 month** |
| --- | --- | --- | --- |
| 1. Social avoidance/anxiety | 29 | 36 | 21 |
| 2. Psychological agitation | 18 | 15 | 12 |
| 3. Daily Activities | 22 | 11 | 11 |
| 4. Need for assistance/positioning | 15 | 11 | 12 |
| 5. Positive Impact | - | - | - |
| 6. Need for intervention | 3 | 9 | 1 |
| 7. Social Embarrassment | 7 | 2 | 3 |
| 1. Social avoidance/anxiety | 0 | 0 | 1 |
| 2. Psychological agitation | 1 | 1 | 4 |
| 3. Daily Activities | 6 | 4 | 2 |
| 4. Need for assistance/positioning | 2 | 1 | 1 |
| 5. Positive Impact | - | - | - |
| 6. Need for intervention | 0 | 0 | 0 |
| 7. Social Embarrassment | 0 | 0 | 0 |
| 1. Social avoidance/anxiety | 19 | 15 | 21 |
| 2. Psychological agitation | 7 | 9 | 12 |
| 3. Daily Activities | 17 | 15 | 12 |
| 4. Need for assistance/positioning | 14 | 11 | 9 |
| 5. Positive Impact | - | - | - |
| 6. Need for intervention | 6 | 3 | 5 |
| 7. Social Embarrassment | 6 | 6 | 9 |
| 1. Social avoidance/anxiety | 23 | 21 | 20 |
| 2. Psychological agitation | 12 | 10 | 10 |
| 3. Daily Activities | 13 | 10 | 9 |
| 4. Need for assistance/positioning | 3 | 6 | 6 |
| 5. Positive Impact | - | - | - |
| 6. Need for intervention | 2 | 1 | 1 |
| 7. Social Embarrassment | 7 | 7 | 5 |
| 1. Social avoidance/anxiety | 27 | 7 | 4 |
| 2. Psychological agitation | 20 | 5 | 0 |
| 3. Daily Activities | 13 | 5 | 7 |
| 4. Need for assistance/positioning | 13 | 3 | 2 |
| 5. Positive Impact | - | - | - |
| 6. Need for intervention | 18 | 2 | 0 |
| 7. Social Embarrassment | 14 | 2 | 0 |
| 1. Social avoidance/anxiety | 43 | 7 | 7 |
| 2. Psychological agitation | 20 | 3 | 3 |
| 3. Daily Activities | 15 | 0 | 0 |
| 4. Need for assistance/positioning | 16 | 0 | 0 |
| 5. Positive Impact | - | - | - |
| 6. Need for intervention | 9 | 0 | 0 |
| 7. Social Embarrassment | 20 | 4 | 4 |
| 1. Social avoidance/anxiety | 2 | 0 | 0 |
| 2. Psychological agitation | 4 | 0 | 0 |
| 3. Daily Activities | 9 | 0 | 1 |
| 4. Need for assistance/positioning | 5 | 0 | 0 |
| 5. Positive Impact | - | - | - |
| 6. Need for intervention | 4 | 0 | 0 |
| 7. Social Embarrassment | 8 | 2 | 0 |
| 1. Social avoidance/anxiety | 10 | 3 | 3 |
| 2. Psychological agitation | 6 | 2 | 4 |
| 3. Daily Activities | 2 | 4 | 4 |
| 4. Need for assistance/positioning | 2 | 3 | 3 |
| 5. Positive Impact | - | - | - |
| 6. Need for intervention | 0 | 0 | 0 |
| 7. Social Embarrassment | 2 | 2 | 3 |

Note the final timepoint was excluded due to low completion rate
